# Decreased Evoked Slow-Activity After tDCS in Disorders of Consciousness

**DOI:** 10.3389/fnsys.2020.00062

**Published:** 2020-09-25

**Authors:** Armand Mensen, Olivier Bodart, Aurore Thibaut, Sarah Wannez, Jitka Annen, Steven Laureys, Olivia Gosseries

**Affiliations:** ^1^Coma Science Group, GIGA Consciousness, University of Liège, Liège, Belgium; ^2^Department of Neurology, University Hospital of Liège, Liège, Belgium; ^3^Centre du Cerveau^2^, University Hospital of Liège, Liège, Belgium; ^4^Neuromodulation Center, Spaulding Rehabilitation Hospital, Harvard Medical School, Boston, MA, United States

**Keywords:** disorders of consciousness, transcranial direct current stimulation, transcranial magnetic stimulation, electroencephalography, bistability, slow activity, diagnosis, treatment

## Abstract

Due to life-saving medical advances, the diagnosis and treatment of disorders of consciousness (DOC) has become a more commonly occurring clinical issue. One recently developed intervention option has been non-invasive transcranial direct current stimulation. This dichotomy of patient responders may be better understood by investigating the mechanism behind the transcranial direct current stimulation (tDCS) intervention. The combination of transcranial magnetic stimulation and electroencephalography (TMS-EEG) has been an important diagnostic tool in DOC patients. We therefore examined the neural response using TMS-EEG both before and after tDCS in seven DOC patients (four diagnosed as in a minimally conscious state and three with unresponsive wakefulness syndrome). tDCS was applied over the dorsolateral prefrontal cortex, while TMS pulses were applied to the premotor cortex. None of the seven patients showed relevant behavioral change after tDCS. We did, however, find that the overall evoked slow activity was reduced following tDCS intervention. We also found a positive correlation between the strength of the slow activity and the amount of high-frequency suppression. However, there was no significant pre-post tDCS difference in high frequencies. In the resting-state EEG, we observed that both the incidence of slow waves and the positive slope of the wave were affected by tDCS. Taken together, these results suggest that the tDCS intervention can reduce the slow-wave activity component of bistability, but this may not directly affect high-frequency activity. We hypothesize that while reduced slow activity may be necessary for the recovery of neural function, especially consciousness, this alone is insufficient.

## Introduction

Disorders of consciousness (DOC) have become a growing clinical concern with the advance of medical technologies, which have limited the fatal consequences of severe brain injury events. Distinctions have been made between the levels of recovery from coma, unresponsive wakefulness syndrome/vegetative state (UWS), minimally conscious state (MCS), to emergence from MCS (EMCS) (Giacino et al., 2002; Laureys et al., 2010). Each step is associated with increasing signs of awareness of one’s self or the patient’s environment. While in UWS the patient is awake, no such behavioral signs can be reliably found in the patients within this group. Reliable evidence of awareness then characterizes the patient as MCS, which has been subsequently divided into MCS− and MCS+ depending on the absence or presence of language processing (Bruno et al., 2011). Further signs of functional communication or object use transition the patient to EMCS (Giacino et al., 2002). Patients who are unresponsive at the bedside (i.e., UWS) may, however, present similar brain activity to patients in MCS, and they are referred as non-behavioral MCS or MCS^∗^ (Gosseries et al., 2014b; Stender et al., 2014). The development of accurate diagnostic tools to distinguish these states has been key in the understanding of the basic mechanisms of neural activity and the clinical treatment options and also has important ethical and legal implications (Gosseries et al., 2014a; Fins, 2016).

Transcranial direct current stimulation (tDCS) has recently shown promise as a non-invasive, non-pharmaceutical intervention in DOC patients (Thibaut et al., 2019). This technique of passing a small electric current through two electrodes attached to the scalp has been well established to modulate the neural activity of the underlying networks (Nitsche and Paulus, 2000). One stimulation site in particular, the left dorsolateral prefrontal cortex (DLPFC, with the cathode over the right supraorbital area), has been shown to be effective in a number of studies (Angelakis et al., 2014; Thibaut et al., 2014, 2017, 2018a; Naro et al., 2015; Estraneo et al., 2017; Martens et al., 2018; Cavinato et al., 2019; Wu et al., 2019; Hermann et al., 2020). For instance, of 30 MCS patients undergoing just a single session of tDCS, 13 showed new signs of conscious behavior following the intervention (Thibaut et al., 2014). More recently, it was shown that repeated sessions of the same protocol over 5 days significantly improved scores on the Coma Recovery Scale – Revised (CRS-R) in 9 of 16 chronic MCS patients, even 1 week after the end of the stimulation protocol (Thibaut et al., 2017).

While the evidence for clinical improvement is mounting, we nonetheless lack a plausible mechanism for these changes. For over a decade now, the level of consciousness can be probed using single pulses of transcranial magnetic stimulation (TMS), with the neural effects of this stimulation captured using electroencephalography (EEG). This TMS-EEG combination has been shown to be an effective discriminator of conscious levels between participants in sleep and wake conditions (Massimini et al., 2005), including periods of dreaming within rapid and non-rapid eye movement sleep (Massimini et al., 2010; Nieminen et al., 2016; Lee et al., 2019). The approach has also differentiated the effects on (un)conscious level in distinct anesthetic agents (Sarasso et al., 2015; Darracq et al., 2018). In the clinical diagnostic setting, the neural response from the TMS pulse has been quantified into the perturbational complexity index (PCI), where values above an empirically defined cutoff of 0.31 can accurately stratify patients into the same clinical categories made with behavioral assessments (Casali et al., 2013; Casarotto et al., 2016; Bodart et al., 2017). Changes to the complexity of a neural signal can theoretically be achieved in a number of ways, yet two principal mechanisms have been identified under the umbrella term “bistability” (Pigorini et al., 2015; D’Andola et al., 2018). Firstly, low complexity states found in deep sleep, anesthesia, and DOC show slow activity indicative of a neural “down-state,” associated with hyperpolarization and the cessation of neural firing. Accompanying this slow wave is a relative suppression of high-frequency neural activity. Following the slow wave, spontaneous neural activity might resume, yet this is no longer causally locked to the initial stimulus. This break in causal influence is thought to underlie the loss of consciousness (Pigorini et al., 2015; Tononi et al., 2016). With this *a priori* mechanistic approach, we aimed to directly examine the features of bistability using the well-established TMS-EEG methodology to perturbing neural activity, both before and after tDCS, in a set of DOC patients.

## Materials and Methods

### Participants and Diagnostic Assessments

Twenty-one patients with chronic DOC were enrolled in this study at the University Hospital of Liege. Thirteen patients did not complete the entire study due to having no brain responses to TMS-EEG prior to tDCS, significant TMS artifacts or muscle artifacts, patients moving too much, and/or technical issues. Eight chronic DOC patients thus successfully completed the whole study, but one participant was excluded from the analyses due to noisy data. Inclusion criteria were that the patient be over 18; diagnosed as UWS or MCS based on repeated assessments with the CRS-R (Giacino et al., 2004) performed by trained and experienced clinicians; had no contraindications for MRI or TMS (i.e., no history of epilepsy, metallic implants, or external shunts); and no history of other neurological or psychiatric problems. This study was approved by the Ethics Committee of the Faculty of Medicine of the University of Liège. Legal guardians of the patients were informed about the study and signed a written informed consent sheet.

### TDCS and TMS Acquisitions

Patients underwent an established TMS-EEG protocol, as previously published (Rosanova et al., 2012), before and after anodal tDCS on the left DLPFC (see Thibaut et al., 2014). tDCS was performed and lasted 20 min at 2 mA with the cathode on the right supra-orbitofrontal area using the DC Stimulator PLUS (NeuroConn, Germany). The TMS target was the medial premotor cortex (just outside the primary effect location of the tDCS), for 400 trials separated by random intervals between 2 and 3 s using a figure-of-eight coil (Focal Bipulse, Nexstim Plc, Finland). Navigation on patients’ 3D T1 MRI image allowed avoiding stimulating over brain lesions (Gosseries et al., 2015). Ultimately, five patients were stimulated over the left premotor cortex while two over the right premotor cortex due to brain lesion location. Resting-state EEG was also acquired before the first TMS-EEG and after the last TMS-EEG session in each patient. Experimenters encouraged the patients to remain awake with their eyes open during the entire time period of recordings. If the patients were unable to do this, breaks were taken and/or a standard arousal protocol was performed (Giacino et al., 2004). This arousal protocol is part of the CRS-R and uses deep pressure stimulation, starting from the facial musculature to the toes on both sides of the body. The muscles are firmly grasped between the thumb and forefinger and are “rolled” back and forth several times. This helps in prolonging the length of time the patient maintains arousal (i.e., eye opening).

### EEG Recording and Preprocessing

EEG was recorded using a TMS-compatible amplifier, with a 60-channel, low-profile cap at 1,450-Hz sampling rate (eXimia, Nexstim Plc, Finland). The raw EEG data was imported into Matlab (version 2017a, MathWorks, Natick Inc., Boston) and processed using EEGLAB (Delorme and Makeig, 2004) and custom, open-source, scripts^1^. An example of the TMS-evoked activity after each preprocessing step is shown in Supplementary Figure 1. A first-order high-pass filter at 0.1 Hz was applied to remove any DC drift. Then, a notch filter (Butterworth 48–52 Hz, sixth order) was applied to remove any power mains noise. This was followed by a bandpass filter (Butterworth 0.5–45 Hz, eighth order). Trials were then created 800 ms before and after each TMS pulse. Bad channels were manually detected and removed (*Mean* = 5.86, *SD* = 2.93). Trials with excessive muscle artifacts were also manually selected and removed, as well as trials with eye movements around the time of the pulse (*Mean* = 113.0 of 400, *SD* = 49.82).

Independent component analysis was then performed with a reduction of data to the first 25 principal components (as implemented in EEGLAB’s “*binica*” function). Twenty-five were selected so that each patient had the same number of possible components, regardless of the number of bad channels that were previously removed. Moreover, a reduction in the number of components made the assumption of sufficient data points for adequate component convergence more tenable. Independent components were visualized according to the spatial topography of their weights, trial-by-trial activity, mean evoked activity, spectral transformation, and the component time series. Furthermore, we could explore, online, the effect of a single component removal (or re-addition) on the channel time series. Clear eye blink/movement components were principally identified by their topography and removed. Muscular artifacts were identified by their higher-frequency spectral power, generally lateral topography, and no clearly evoked activity (see Supplementary Figure 2 for examples). Residual activity directly related to the TMS pulse was identified through their mean evoked activity confined within 20 ms of the event marker. After component removal, activity of the missing channels was recalculated using the spline interpolation approach, and the data was then referenced to the average activity of all channels at each sample.

### TMS-EEG Analysis

Event-related potentials were analyzed at the individual level by comparing pre-tDCS trials with post-tDCS trials using *t*-tests for each channel and time point. This mass univariate approach was corrected for multiple comparisons using a threshold-free cluster enhancement (TFCE) technique followed by a maximum permutation approach (Mensen and Khatami, 2013; Pernet et al., 2015). The same approach was used at the group level using paired *t*-tests for each participant between pre- and post-tDCS on the mean event-related potential (ERP).

Parameters of bistability were assessed in line with those previously used for intracranial EEG (Pigorini et al., 2015). Slow activity was defined as the evoked activity found after filtering the original data between 0.5 and 6 Hz (fourth-order Butterworth filter). Event-related spectral perturbation (ERSP) was calculated using EEGLAB’s newtimef function, which uses Morlet wavelets to decompose each trial into the power at increasing frequency bands and baseline corrects the evoked activity for each trial using the baseline period from −400 to −200 ms (low frequencies will leak temporally into the baseline period, so −200 ms is a safe period). This was then converted to decibels (using log10) and then averaged across all trials. From this, the high frequencies were considered in the range of 20–45 Hz. Both high-frequency activation and suppression can be calculated by examining the fifth lowest percentile of activity below zero for suppression and the 95th highest percentile above zero for activation across the range of high frequencies and time points within defined windows described below.

The individual nature of the TMS response made point-to-point analysis unreliable. However, specific aspects of the response could be summarized across the topography and time series for each participant and effectively compared at the group level. Given the strong prior hypotheses on the importance of slow activity, we summarized this effect by taking the minimum (i.e., largest negative amplitude) activity for the slow-wave, bistability measure for each participant in three time windows. The baseline period ran from −400 to −100 ms, the maximum early slow response was taken between 0 and 200 ms, while the late response was taken between 200 and 500 ms. Main effects of the individual terms were assessed using a likelihood ratio test on the complete model vs. the reduced model without that particular term. We explored the consistency of any effect found by examining the minimum 50–95% percentiles of activity; not only the absolute minimum response from each participant. This summary data was assessed for statistically significant differences by a linear mixed model with time window, tDCS condition, and behavioral diagnosis as fixed factors and participant as a random effect.

### Resting-State EEG Analysis

Each participant had two resting-state recordings, one before and one after tDCS, each lasting between 5 and 8 min. Preprocessing proceeded along identical lines as with the TMS-EEG recordings, with a few exceptions: the data was kept as a single continuous recording, and artifacts were manually marked and excluded from the independent component analysis (ICA) and all further analyses. Individual slow waves were detected using an open-source MATLAB-based toolbox^2^ (Mensen et al., 2016). In a first stage, the negative envelope of all channels was calculated and then bandpass filtered between 0 and 4 Hz (Chebyshev type 2). The negative envelope is given by taking the mean of the most negative three channels at any given time point (default toolbox setting). This new canonical time series was examined for slow waves by checking for the amplitude and duration of waves between the downward and subsequent upward zero crossings. The duration criterion was kept to its default between 250 and 1,250 ms, while the amplitude criterion was set to an absolute value of 15 μV. The amplitude criterion is lower than that in previous analyses for three reasons: average reference tends to reduce amplitudes, especially in the case of more global waves over more channels; the high-pass filter of the negative envelope will baseline shift the entire time series positive; and we can include the individual wave amplitude in the analysis to see whether this had a significant interaction with the other parameters. Several parameters were taken from each detection and examined for pre- vs. post-tDCS effects: wave amplitudes; globality (as percent of channels involved in the slow wave); its duration (essentially the inverse of its base frequency); both the negative and positive peaks; and, finally, the time since the previous wave (an indicator of wave incidence).

## Results

### Clinical Results

The data of seven patients with DOC (four females; mean age of 34.7 ± 10.5 years, mean time since the event 70.9 ± 72 weeks, range 13–200) were analyzed for this study. Four of the patients suffered a traumatic brain injury, two had a hemorrhagic stroke, and one suffered damage from anoxia. Three patients were diagnosed MCS + (i.e., relative preservation of language function), one MCS− (i.e., signs of consciousness not related to language such as fixation), and three UWS (i.e., only reflex behaviors). However, two UWS patients show brain activity compatible with consciousness using TMS-EEG, positron emission tomography, and/or functional magnetic resonance imaging and were thus considered as MCS^∗^. No significant behavioral changes were observed in any of the patients after tDCS. None of the patients showed a change of diagnosis or new signs of consciousness after the tDCS session. Table 1 reports the individual demographical and clinical data.

**TABLE 1:** Demographical and clinical data of patients with disorders of consciousness (DOC).

Patients	Diagnosis pre-tDCS	Diagnosis post-tDCS	Gender	Age	Etiology	Time since injury (weeks)	Structural imaging	Treatment
1	UWS	UWS	F	25	TBI	33	Right basal ganglia hemorrhagic lesion, DAI maximal in both frontal and mesiotemporal areas	Lamictal
2	MCS +	MCS +	F	26	TBI	145	Right frontal CSF shunt, hydrocephaly, signs of ancient right subdural hematoma	Amantadine
3	MCS +	MCS +	F	32	TBI	200	DAI, global atrophy, no focal lesions	/
4	MCS−	MCS−	M	54	Anoxic	23	Global atrophy	Keppra, amantadine
5	UWS (MCS*)	UWS	M	40	TBI	45	Right frontal CSF shunt, DAI in both frontal lobes, lesions on the parieto-occipital junction on both sides, pre-rolandique lesions more on the left side, left frontal superior gyrus, pons, moderate global atrophy	Amantadine, diazepam, baclofen intrathecal, gabapentine
6	MCS +	MCS +	M	39	Stroke	37	Right MCA hemorrhagic lesion with incidental hygroma, basal ganglia involvement on the left side	Keppra, baclofen, sirdalud
7	UWS (MCS*)	UWS	F	27	Stroke	13	Right temporo-parieto-occipital, left orbitofrontal, thalamic and mesencephalic lesions. Right shunt	Amantadine, zolpidem (to sleep), Tramadol

Patients 5 and 7 were diagnosed MCS* based on partial metabolic preservation of the frontoparietal network (Stender et al., 2014) and high values of the perturbational complexity index (PCI) (Casali et al., 2013). Patient 7 also showed robust and specific brain activity during active paradigms (Monti et al., 2010) with functional magnetic resonance imaging. tDCS, transcranial direct current stimulation; UWS, unresponsive wakefulness syndrome; MCS, minimally conscious state; M, male; F, female; TBI, traumatic brain injury; DAI, diffuse axonal injury; CSF, cerebrospinal fluid; MCA, middle cerebral artery.

### Individual-Evoked Potentials

**FIGURE 1:**
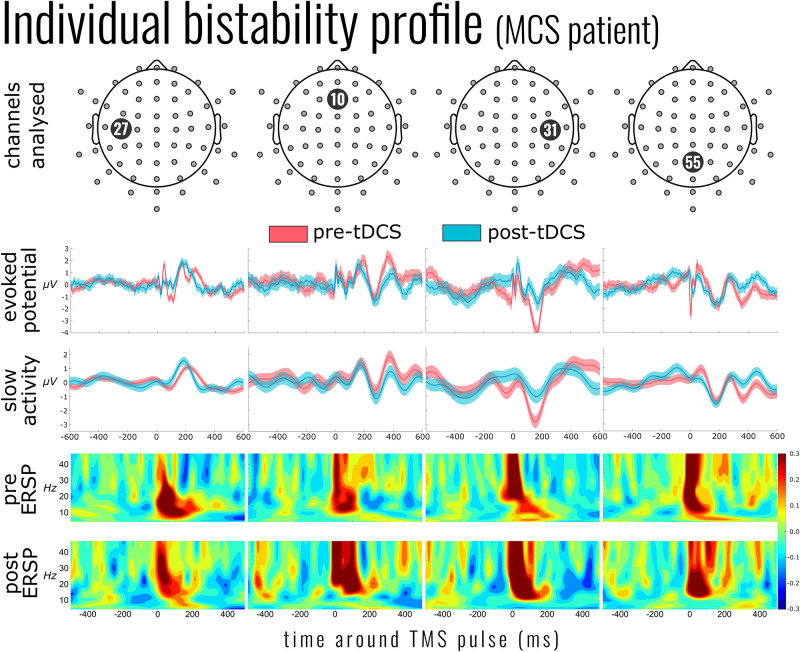
Individual bistability profile of a patient in minimally conscious state (MCS; patient 6). *Top panel* indicates the exemplar channels 27, 10 [channel in the center of the supposed effect area of the transcranial direct current stimulation (tDCS)], 31 [nearest channel to the transcranial magnetic stimulation (TMS) pulse], and 55. The evoked potential panel shows the average electroencephalography (EEG) activity 600 ms before and after the single TMS pulses (mean of 267 pulses pre-tDCS and 220 post-tDCS). Below this is the mean slow activity, a low-pass-filtered signal of the TMS-evoked potential. The slow activity profile of channel 31 (*third column*) shows the reduction of slow activity after tDCS. The *two panels below* show the event-related spectral perturbation before and after tDCS intervention. *Yellow* to *red* indicates an increase in the power of the corresponding frequency (compared to the mean baseline activity), while *shades of blue* indicate a suppression of those frequencies.

All participants showed some significant changes between the pre- and post-tDCS trials at the individual level (see Figure 1 for an example). The pattern of these changes, however, was highly variable between patients as none shared a peak significant channel or time point. Nor was any single channel or time point significantly different for even six of the seven patients (only 15 channels shared significant differences for five of seven participants). This non-overlapping of significant individual differences was confirmed in the group analysis comparing the average evoked potentials, which found no significant changes to the ERPs at the group level [peak channel E9 at 33 ms, corresponding approximately to channel F2 in the 10/20 system; *T*(6) = 12.512, *p* = 0.365].

### Bistability Measures

**FIGURE 2:**
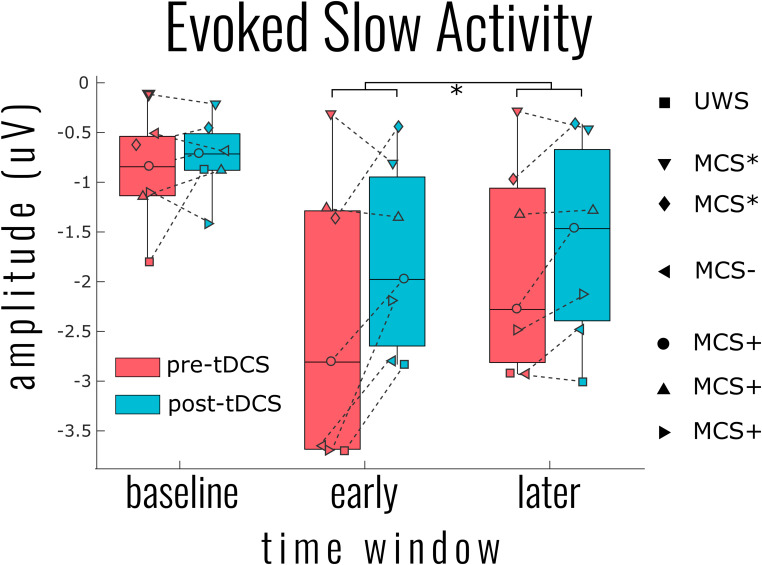
Group results for the effect of transcranial magnetic stimulation (tDCS) on the evoked slow activity. The single point per individual indicates the most negative 5% of activity across all channels within the indicated time windows: baseline (−400 to −100 ms), early (0–200 ms), and later (200–400 ms). This percentile approach within time windows was necessary to generalize the patterns of results over all patients given the highly individualized responses. Linear mixed model analysis indicated a significant reduction of slow activity (i.e., smaller negative amplitude) at both the early and later time windows [χ^2^(2) = 12.141, *p* = 0.002]. A significant tDCS effect was found for all negative percentiles across channels from 5% (shown) to 50%.

Two distinct aspects of the EEG response were derived to capture proxies of cortical bistability: the slow response and high-frequency activity. The results for the slow responses showed that, at the bottom fifth percentile of each individual patient’s response, there was a significant attenuation of the slow activity after tDCS at both the early and later time windows [mean ERP amplitude change = 0.45 ± 0.56 μV, χ^2^(2) = 12.141, *p* = 0.002] (see Figure 2). There was also a main effect of the time window with a smaller (less negative) slow response at the later time [χ^2^(2) = 8.308, *p* = 0.0157], yet no interaction with the tDCS condition [χ^2^(1) = 2.021, *p* = 0.155]. This pattern of results was significant from the fifth percentile to the 45th lowest percentile response, yet with steadily decreasing variance being accounted for (model *R*^2^ from 0.87 down to 0.66). At the individual channel level, there was no significant decrease in slow activity after tDCS, however, the general pattern of results indicated that, for any given patient, the decrease was most likely to be found in posterior channels. Information about the DOC classification of the patients (UWS or MCS) was not a significant predictor of slow wave activity [χ^2^(4) = 3.847, *p* = 0.427].

**FIGURE 3:**
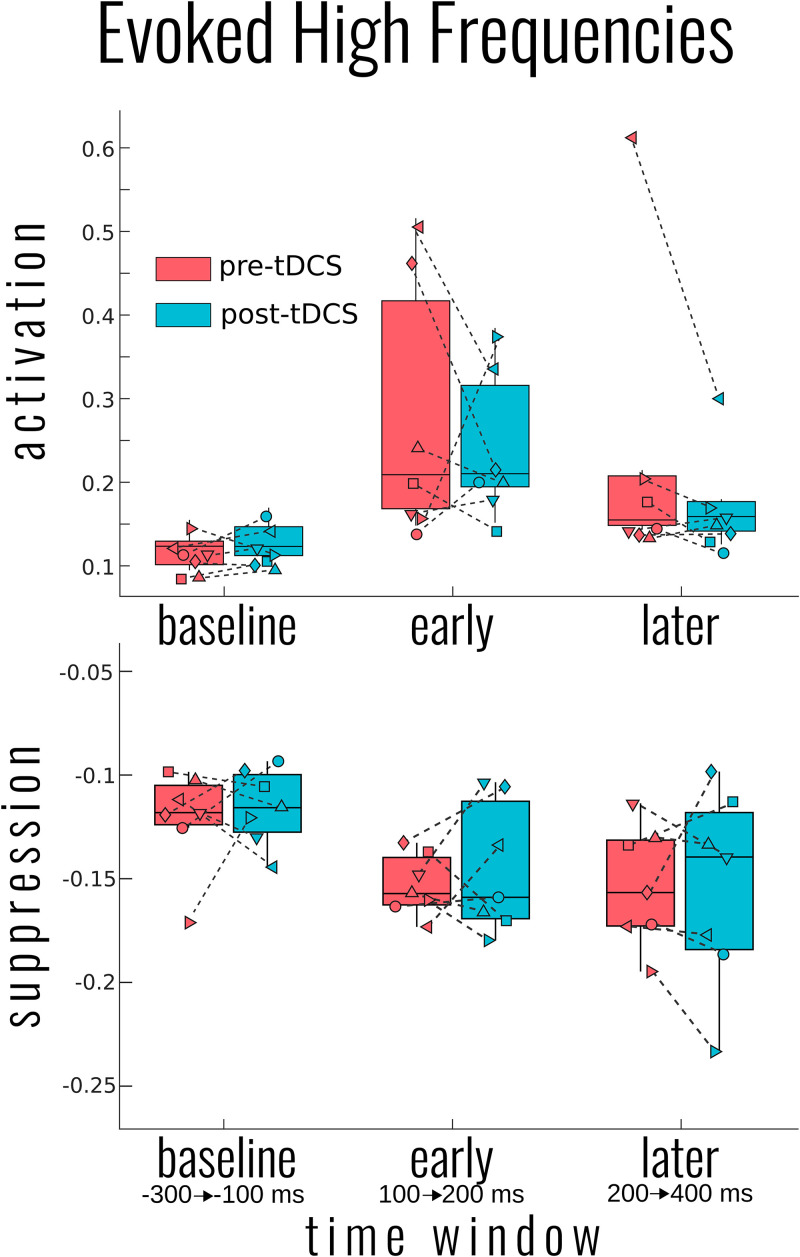
Group results for the effect of transcranial magnetic stimulation (tDCS) on the evoked high-frequency power. Activation (**top**) and suppression (**bottom**) of high frequencies (20–50 Hz) for each participant and group average in box plots as measured by the bottom and top 5% of the event-related spectral perturbation over each of the indicated time windows. No significant differences in pre- to post-tDCS were found for either high-frequency measure. Each patient’s disorders of consciousness (DOC) classification can be found in Figure 2 above.

High-frequency activity was examined in a similar approach to slow-wave activity. As Figure 1 (ERSP) exemplifies, the time period around the TMS pulse was saturated in power across a large range of frequencies; we therefore adjusted the early time period between 100 and 200 ms. As Figure 3 illustrates, there was a significant activation [χ^2^(4) = 17.090, *p* = 0.002] and suppression [χ^2^(4) = 18.053, *p* = 0.001] of high frequencies evoked by the TMS pulse compared to baseline. However, unlike the slow activity, we found no significant differences before and after tDCS in either activation [mean activation change = −0.04 ± 0.13, χ^2^(2) = 1.999, *p* = 0.368] or suppression [mean suppression change = 0.003 ± 0.03, χ^2^(3) = 0.231, *p* = 0.973]. Information on the DOC diagnosis of individual patients significantly improved the overall model for high-frequency suppression [χ^2^(6) = 15.397, *p* = 0.017], but not activation [χ^2^(6) = 4.987, *p* = 0.546].

We also examined the relationship between the slow response and high-frequency suppression directly. We found that these two measures significantly correlated with one another [*r* = 0.657, χ^2^(1) = 13.664, *p* < 0.001]. Furthermore, once the slow activity was included in the mixed model with the tDCS condition, there was a significant interaction between these two factors on the early time window [χ^2^(1) = 7.092, *p* = 0.008]. As Supplementary Figure 5 illustrates, prior to tDCS, the relationship between these two factors was positive, but weak and non-significant [χ^2^(1) = 1.052, *p* = 0.305]. After tDCS, the more the slow activity was reduced, the less the high frequency was suppressed [χ^2^(1) = 7.715, *p* = 0.006]. This interaction between slow activity and condition was not the case for the later time window [χ^2^(1) = 0.215, *p* = 0.643], nor for the high-frequency activations [χ^2^(1) = 0.301, *p* = 0.583]. Here, again, we found a global effect of DOC diagnosis [χ^2^(6) = 14.507, *p* = 0.025] on the prediction of high-frequency suppression even when slow activity was also included.

### Resting State

Given the effects of tDCS on slow activity, we further examined patients’ resting-state recordings. For two MCS patients, no slow waves were detected using the current criteria, and so the analysis focused on the five remaining participants. A total of 962 waves were detected in all recordings, 608 in resting state prior to tDCS and 354 after tDCS. As with the TMS-EEG response, at the individual participant level, several distinct parameters could be found to be significantly different between the two conditions (see Supplementary Figure 4 for an example). At the group level, we found main effects on two primary slow-wave parameters. After tDCS, the positive slope of the wave was significantly reduced [χ^2^ = 10.116, *p* = 0.002]. This effect remained when the wave amplitude was controlled for [χ^2^ = 10.298, *p* = 0.001]. Yet while we found the expected correlation with the negative slope (*r*^2^ = 0.764, χ^2^ = 996.830, *p* < 0.001), tDCS did not seem to systematically affect this parameter at the group level (χ^2^ = 1.490, *p* = 0.222). Time since last wave, effectively the inter-wave temporal spacing, was also found to be significantly longer after tDCS (estimated difference of 1.287 s; χ^2^ = 18.524, *p* < 0.001). Stepwise addition of the other parameters to this model revealed no further interactions or unique effects beyond the main effect of time.

## Discussion

This study sought to examine the effect of prefrontal tDCS on cortical bistability in a small sample of DOC patients. The combination of TMS-EEG is regularly used to aid in the classification of DOC patients, specifically in the differentiation of UWS and MCS, by perturbing the state of the brain and capturing the causal influence (Napolitani et al., 2014; Sarasso et al., 2014). Firstly, none of the seven patients demonstrated any novel signs of consciousness following tDCS. Given that previous studies have shown that around half of MCS patients showed some improvement, this was somewhat unexpected (Thibaut et al., 2014; Estraneo et al., 2017). However, with our focus on the TMS-EEG measures, behavioral assessments were delayed compared to the previous studies and so could have missed acute changes. Another reason for the lack of behavioral changes was likely the high intensity of the entire experimental protocol, lasting between 4 and 6 h, undoubtedly causing fatigue for the patients. Regarding the response to TMS, we found that each patient had relatively unique evoked potentials in response to the TMS pulse, with significant effects of tDCS on the direct spatiotemporal activity at the individual level, but with no consistent, specific effects over all patients. We then examined the two hallmark signatures of cortical bistability: the slow wave and the following suppression of high-frequency activity. We found a significant group-level reduction in slow activity after tDCS, yet with a non-specific spatial profile consistent with individual variability. Furthermore, patients’ slow activity was related to the amount of high-frequency suppression, consistent with the concept of bistability. However, there was no group-level reduction in the amount of suppression or in the activation of high frequencies, suggesting that, while correlated, slow activity and high frequencies can be, and were, independently modulated. The effect of tDCS on slow activity was further supported by demonstrating that the properties of individual slow waves were also significantly altered after tDCS. While their amplitudes remained unchanged, there was a significant reduction in the incidence of slow waves.

Previously, Bai et al. (2017) demonstrated an increase in cortical excitability in both MCS and UWS patients following the same tDCS protocol as used here. Their measure of cortical excitability was the global mean field power (GFP) of the mean EEG response following TMS. Given that most of the power in the EEG signals will indefinitely reside in the lower spectral frequencies (i.e., slow activity), this GFP increase initially seems to counter the decrease of slow activity presented in our study. Therefore, one key difference in these studies is the analytical approaches used. While Bai and colleagues examined more general and widely used metrics, here, we focused on measures of bistability that have a closer theoretical underpinning to the study of consciousness (Pigorini et al., 2015). However, when we reanalyzed our data using GFP (see Supplementary Figure 3), we found an early decrease, but which was not significant. We believe that the key distinction between these projects is the site of the TMS. Bai and colleagues stimulated directly over the left DLPFC, presumably over the region of the cortex directly altered by the tDCS intervention (Bai et al., 2017). On the other hand, we perturbed the premotor cortex, which lies outside the main electric field of the tDCS and, therefore, over areas of the cortex that would have retained more of their physiological responses. Here, we were primarily concerned with electrophysiological measures of conscious awareness and not in the cortical effects of tDCS *per se*. We are therefore confident that perturbation outside the direct influence of the intervention is a more meaningful way to examine the global neural effects. In this regard, it is not unreasonable to expect that, had our TMS site been over the DLPFC, or that of Bai and colleagues been outside this area, we would have found similar results.

It is critical to note that none of the patients we recorded showed a significant behavioral improvement despite various degrees of slow activity modulation. On its surface, this suggests that the reduction of this neural slow activity is insufficient for the recovery of important networks underlying potential behavioral improvement. There are three potential elements at work here, none mutually exclusive. The first is that, despite some reduction in the slow activity, further reduction, i.e., larger clusters of neurons out of the bistable state, is necessary for normal functioning. Secondly, while slow activity can be reduced, this is not enough for the resumption of high-frequency activity associated with the normal functioning of critical networks, and despite their correlation, there is an intermediate step between these elements that is often not directly affected through tDCS. Thirdly, there is an interaction of slow activity and high frequencies within certain brain areas, cortical layers, or across distinct networks that are damaged in certain patients and not others. The latter is certainly the case, to some extent, and likely the most difficult situation to disentangle.

Regarding the resting-state EEG results, we mainly found a significant reduction in the incidence of slow waves after tDCS. In the same line, a recent EEG study reported decreases in the delta band when assessing coherence in patients with DOC after tDCS over the precuneus (Guo et al., 2019). Other EEG studies showed increased frontoparietal coherence in the alpha, beta, and/or theta frequency bands (Bai et al., 2018; Cavinato et al., 2019), along with an increase in P300 amplitude (Zhang et al., 2017) after tDCS over the DLPFC in MCS patients. Comparing tDCS responders to non-responders, increased EEG power, network centrality, and functional connectivity were found in the theta and theta–alpha bands, with larger P300 responses after tDCS (Thibaut et al., 2018b; Hermann et al., 2020).

Thibaut et al. (2018a) present a case study of a patient showing limited signs of consciousness, but whose passive neuroimaging examination showed preserved activity and metabolism. tDCS was able to unveil signs of conscious behavior in this patient. With respect to the results here, tDCS can modulate neural networks, but patients likely already need to be close to the necessary conditions for recovery of function. That is, that key networks need to be structurally preserved, albeit possibly in a bistable state. Modulation of patients’ slow activity might then physiologically be accompanied by a reactivation of the high frequencies necessary for behavior improvement. Future work should aim to preselect patients based on their prior demonstrated responsiveness to tDCS (or lack of), with extensive qualification of the lesions involved (both structurally and functionally). While here we included patients with UWS diagnosis, the previous study that successfully modulated behavior with tDCS was directed toward MCS patients. Here, we present half of the potential results of such a study; that tDCS can indeed modulate bistability, as hypothesized, but this modulation alone is insufficient to improve the behavioral state of patients. We have provided some evidence that the correlated high-frequency suppression may be another key missing element, yet not directly altered by the tDCS. We can only speculate that, in patients who respond behaviorally to the tDCS intervention, the reduction of slow activity is combined with the reemergence of high-frequency activity.

### Limitations and Future Perspectives

Our limited sample size of seven patients is a clear limiting factor in generalizing the results of such a study. However, the demands on the patient and the experimenter of performing multiple sessions of TMS-EEG assessments, in combination with the tDCS intervention, are extraordinarily high. Given the large parameter space of settings and localization of tDCS and the variability in the etiology of patients, we believe that the in-depth characterization of effects at the individual patient level is nevertheless a fruitful path to navigate through this space. A further limitation was the long experimental duration and the non-random nature of the pre–post measures. Patients here may have become fatigued throughout the day, which could have contributed to the pattern of results found here. Further studies could include sham tDCS stimulation, a shorter TMS-EEG protocol, or multiple measures post-tDCS where, presumably, the effects of the intervention may decrease but fatigue would increase to delineate these effects. Future studies may also wish to focus on patients who had previously shown improvement with tDCS to increase the chance of correlating the behavioral improvements to the changes in the bistability measures demonstrated here.

## Conclusion

The present results suggest that a single session of prefrontal tDCS can reduce the slow-wave activity component of bistability, but this may not directly affect the high-frequency activity. We hypothesize that while reduced slow activity may be necessary for the recovery of neural function, especially consciousness, this alone is insufficient as we did not observe significant clinical improvement. Repeated sessions of tDCS may be necessary to induce a behavioral response paralleled with a reduction of slow activity combined with the reemergence of high-frequency activity.

## Data Availability Statement

The raw data supporting the conclusions of this article will be made available by the authors, without undue reservation, to any qualified researcher.

## Ethics Statement

The studies involving human participants were reviewed and approved by the Ethical Committee of the University Hospital of Liege, Liège, Belgium. Written informed consent was provided by the patients’ legal guardians.

## Author Contributions

AM, OB, AT, SL, and OG planned and designed the study. OB, AT, SW, and JA acquired the data. AM and OG analyzed and interpreted the data. AM drafted the manuscript. All the authors revised and approved the manuscript.

## Conflict of Interest

The authors declare that the research was conducted in the absence of any commercial or financial relationships that could be construed as a potential conflict of interest.

## Footnotes

1 https://github.com/CSC-UW/csc-eeg-tools

2 www.github.com/mensen/swa-matlab
