# Supplementary figures and images for: Decreased Evoked Slow-Activity After tDCS in Disorders of Consciousness
Source: Front Syst Neurosci. 2020 Sep 25;14:62. doi: 10.3389/fnsys.2020.00062 (PMC7546425; doi:10.3389/fnsys.2020.00062)

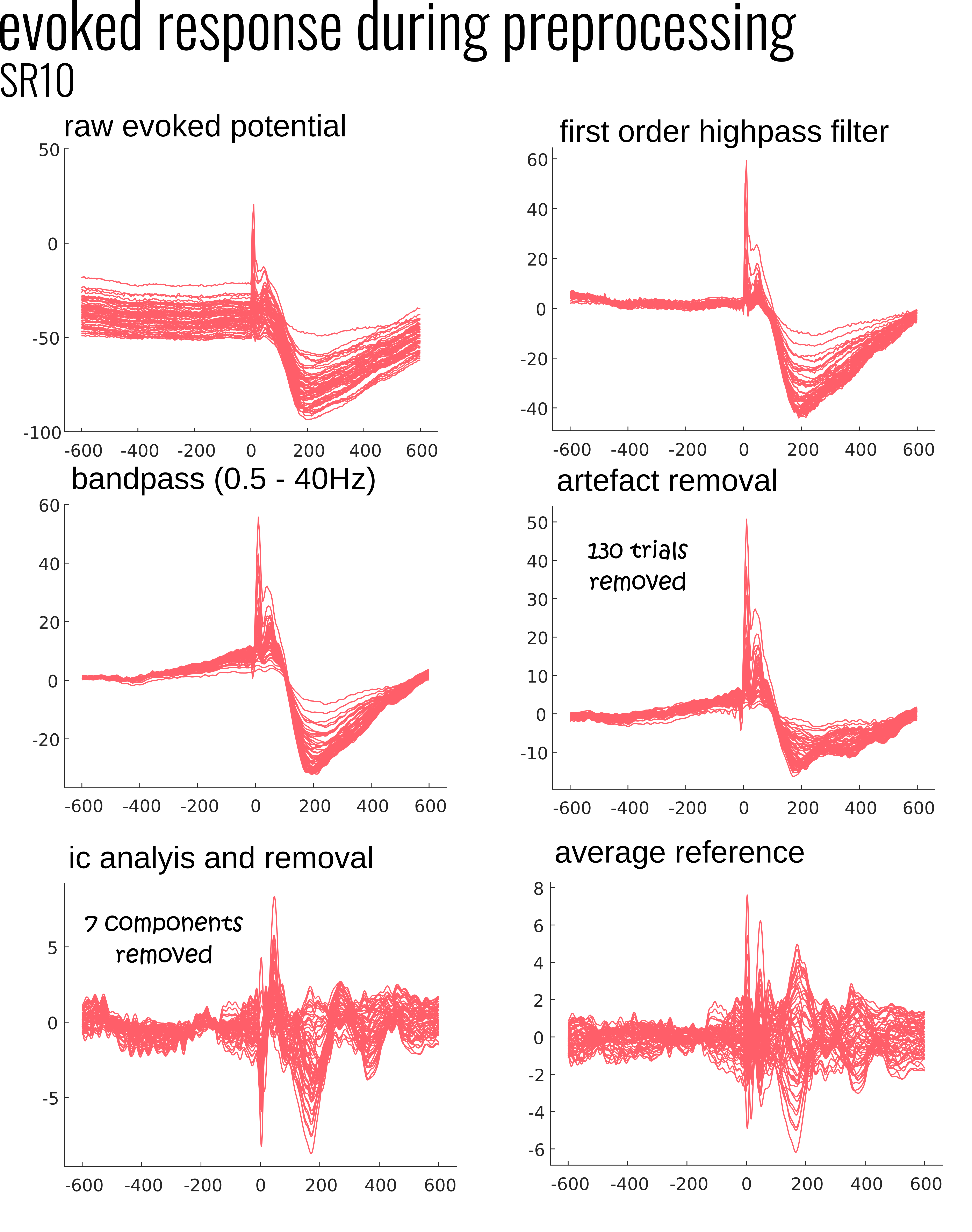

Supplement: Supplementary file 1 [file Image_1.PNG]

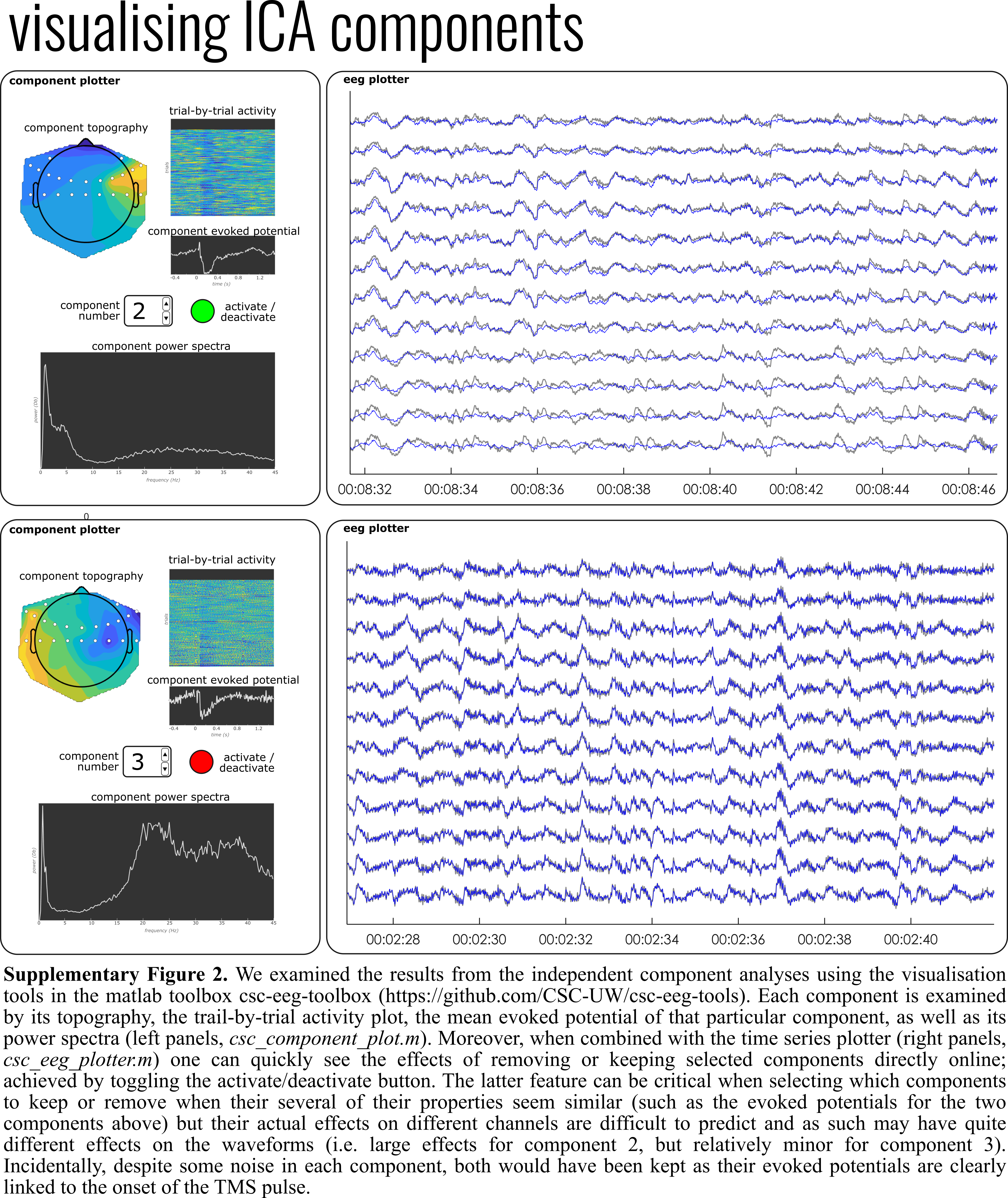

Supplement: Supplementary file 2 [file Image_2.PNG]

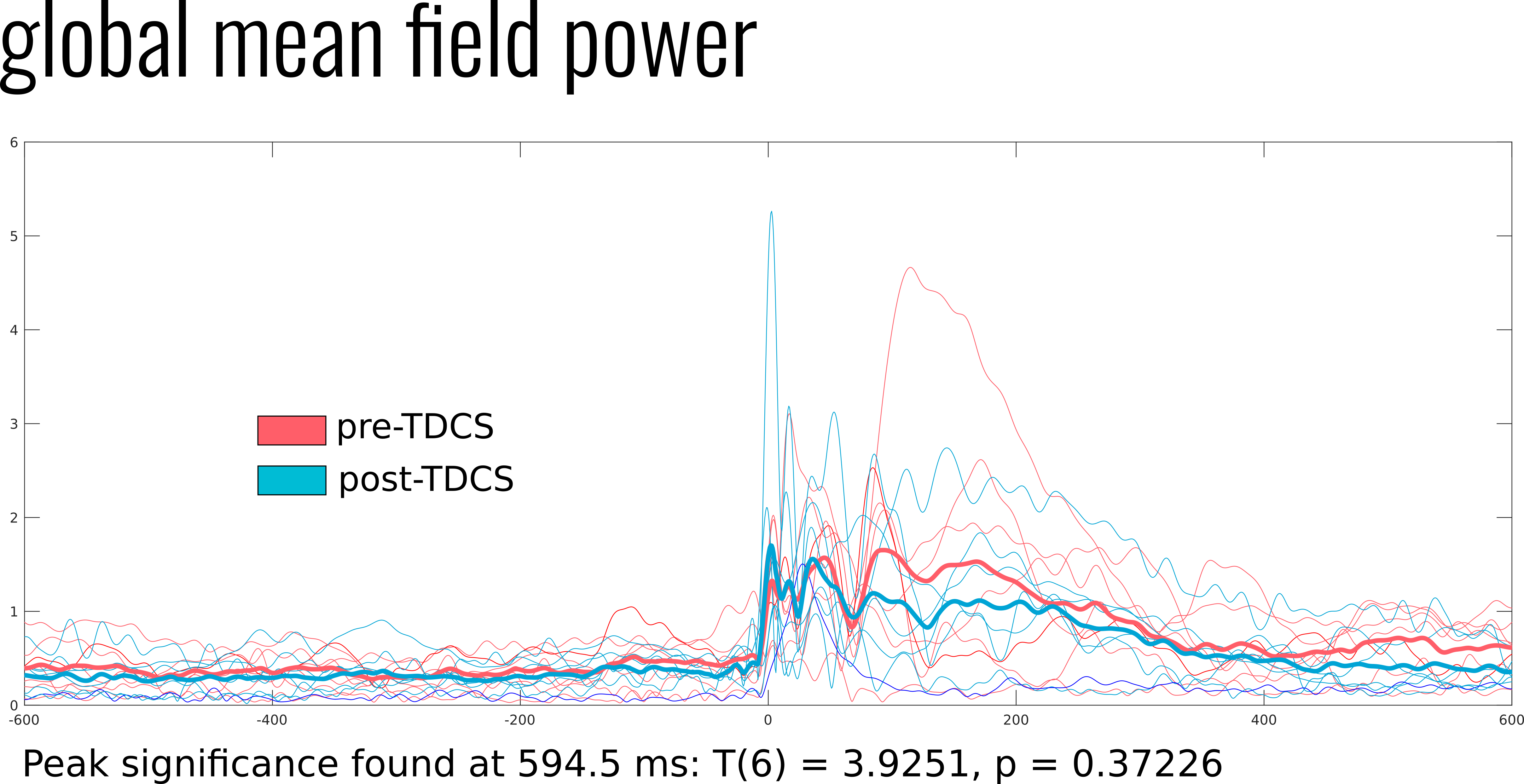

Supplement: Supplementary file 3 [file Image_3.PNG]

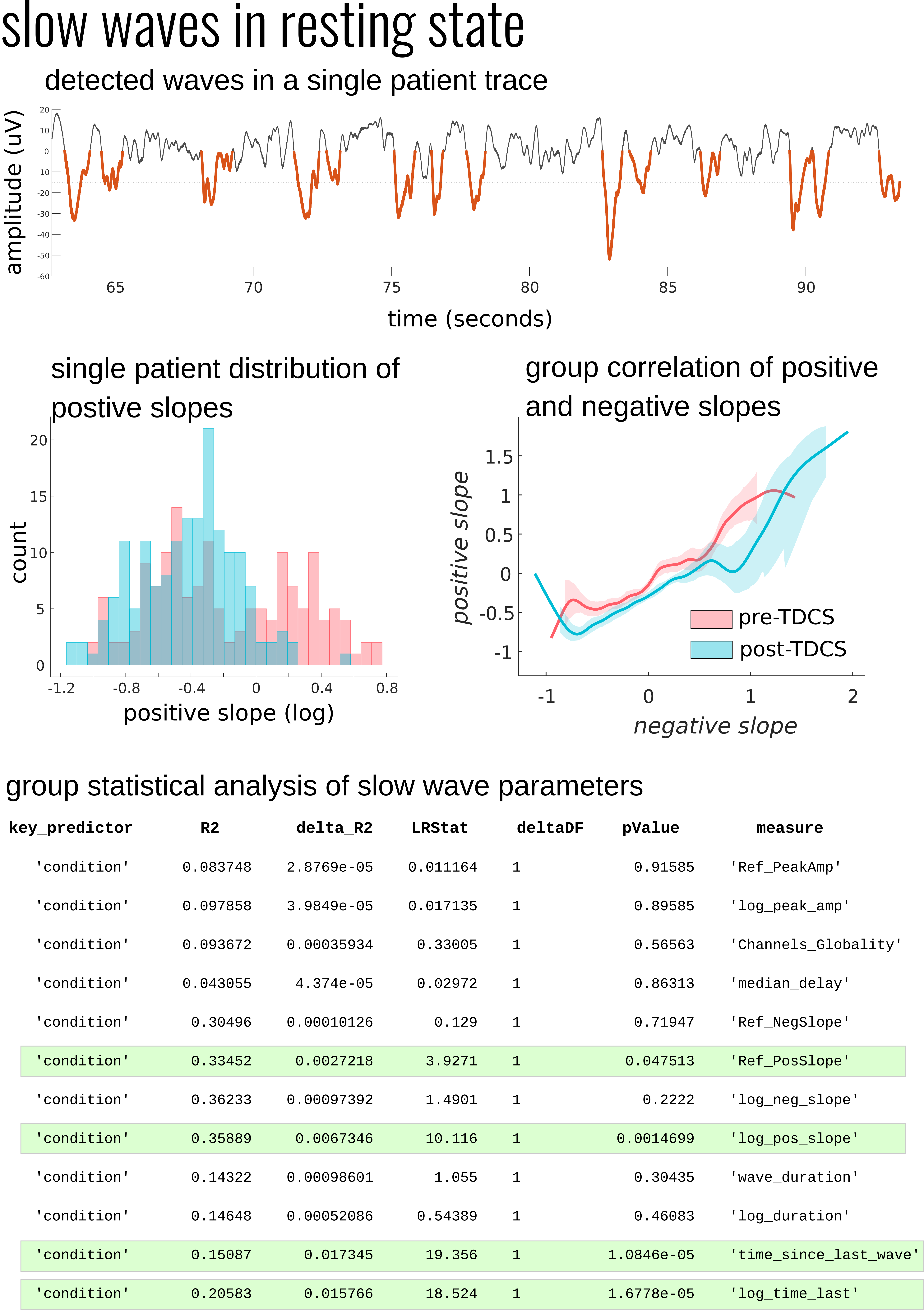

Supplement: Supplementary file 4 [file Image_4.PNG]

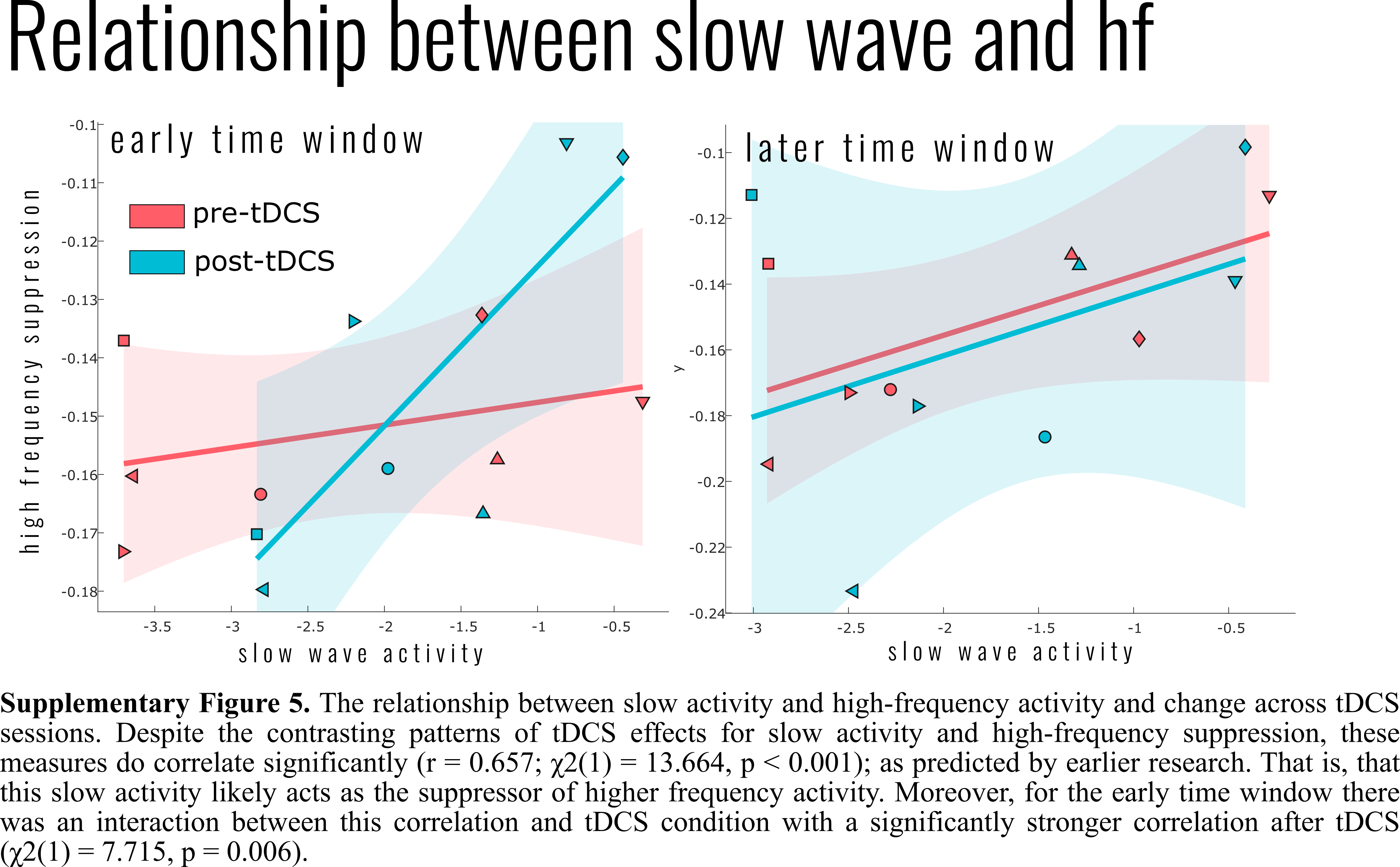

Supplement: Supplementary file 5 [file Image_5.PNG]
